# Heartmate 3 fully magnetically levitated left ventricular assist device for the treatment of advanced heart failure –1 year results from the Ce mark trial

**DOI:** 10.1186/s13019-017-0587-3

**Published:** 2017-04-04

**Authors:** Thomas Krabatsch, Ivan Netuka, Jan D. Schmitto, Daniel Zimpfer, Jens Garbade, Vivek Rao, Michiel Morshuis, Friedhelm Beyersdorf, Silvana Marasco, Laura Damme, Yuriy Pya

**Affiliations:** 1 German Heart Center, Deutsches Herzzentrum Berlin, Augustenburger Platz 1, D-13353 Berlin, Germany; 2 Institute for Clinical and Experimental Medicine, Vídenská 1958/9 Praha 4, Prague, Czech Republic; 3 Hannover Medical School, Carl-Neuberg-Str. 1, 30625 Hannover, Germany; 4 University of Vienna, Waehringer Guertel 18-20, A-1090 Vienna, Austria; 5 Heart Center Leipzig, Struempellstrasse 39, 04289 Leipzig, Germany; 6 Toronto General Hospital, 4N-464 200 Elizabeth St.,, Toronto, ON M5G 2C4 Canada; 7 Herz- und Diabeteszentrum NRW, Georgstr. 11, 32545 Bad Oeynhausen, Germany; 8 University Heart Center Freiburg-Bad Krozingen, Hugstetterstr 55, D-79106 Freiburg, Germany; 9 The Alfred Hospital, 55 Commercial Road, Prahran, VIC 3181 Melbourne, Australia; 10 St. Jude Medical, 11 Da Vincilaan, Zaventem, 1935 Belgium; 11 National Research Cardiac Surgery Center, 010000, 38 Turan St., Astana, Kazakhstan

**Keywords:** HeartMate 3, Magnetic levitation, Heart failure, LVAS

## Abstract

**Background:**

The HeartMate 3 Left Ventricular Assist System (LVAS) (St. Jude Medical Inc., St Paul, MN) with full magnetic levitation allows for wide and consistent blood flow paths and an artificial pulse designed for enhanced hemocompatibility. The HeartMate 3 received market approval in the European Union in 2015 following completion of a multicenter study. After reaching the 6-month study endpoint, patients continue to be followed for 2 years with the 1-year results presented herein.

**Methods:**

A prospective, non-randomized study included adults with advanced heart failure and ejection fraction (EF) ≤ 25%, cardiac index (CI) ≤ 2.2 L/min/m2 while not on inotropes, or inotrope dependent, or on optimal medical management for 45/60 days.

**Results:**

Fifty patients—54% bridge to transplant (BTT) and 46% destination therapy (DT)—were enrolled and implanted with the HeartMate 3. At baseline, 92% of the patients were INTERMACS profiles 2–4, with cardiac index 1.8 + 0.5 L/min/m^2^ and 58% were supported with inotropes. At 1 year, 74% of the patients remain on support, 18% expired, 6% transplanted, and 2% explanted. The adverse events include 12% gastrointestinal bleeding, 16% driveline infections, 18% strokes, and 2% outflow graft thrombosis. There was no hemolysis, pump thrombosis or pump malfunction through 1 year. The six-minute walk test distance increased from a mean of 273 m to 371 m (*P* <0.0001). EQ-5D quality-of-life score increased from a mean of 52.7 to 70.8 (*P* = 0.0006).

**Conclusions:**

The 1-year HeartMate 3 LVAS results show survival and adverse-event profile are similar to other approved devices, with no pump thrombosis or pump failure. Patient’s functional status and quality of life significantly improved over time.

**Trial registration:**

Clinicaltrials.gov registration number: NCT02170363. Registered June 19, 2014.

## Background

Circulatory support with a continuous-flow left ventricular assist device (CF-LVAD) restores circulation, preserves organ function, improves functional status and quality of life, and extends survival for patients with refractory advanced stage heart failure [1–6]. Limitations of current CF-LVAD technology is primarily the result of untoward hematologic responses to the device’s artificial materials and the high shear forces imparted on blood components by the pump [7]. Hemolysis, platelet activation, and cleaving of von Willebrand factor multimeres often occur during CF-LVAD support and are important contributing factors to thrombosis, stroke, and gastrointestinal (GI) bleeding—adverse events that are the primary limitations of CF-LVAD support [8]. Advancing outcomes of CF-LVAD-supported patients entails novel designs that optimize the blood-biomaterial interface and produce blood flow with low shear force for nominal effects on blood components.

The HeartMate 3 Left Ventricular Assist System (LVAS) (St. Jude Medical Inc., St. Paul, MN) was designed for enhanced hemocompatibility by the integration of a fully magnetically levitated rotor for frictionless movement, wide blood flow gaps through the pump for lower shear stress, and textured blood-contacting inner and outer surfaces for establishing a tissue interface with blood [9, 10]. An artificial pulse allows for more complete washing of pump surfaces eliminating stasis as a cause of pump thrombosis and possibly other clinical benefits related to the pulse pressure. Clinical studies evaluating the HeartMate 3 were initiated in June of 2014 at 10 centers in Europe, Australia, Canada, and Kazakhstan [11]. Fifty patients who met the criteria for refractory advanced stage heart failure received the HeartMate 3 in a trial designed to include both bridge to transplant and destination therapy patients [10]. Results of the study demonstrated the performance goal at the 6-month study endpoint was exceeded, with 92% of patients alive with no instances of hemolysis, pump thrombosis or pump failure. Market approval in the European Union was obtained on October 8, 2015. The purpose of this report is to present the 1 year results of the study, and planned follow-up of 2-years for this study group is ongoing [10].

## Methods

The clinical trial protocol, including patient criteria for enrollment, study endpoints, HeartMate 3 LVAS description, and statistical methods have been previously published [10]. In brief, this single-arm, prospective, nonrandomized clinical trial had a primary endpoint of survival at 6 months compared with a performance goal of 88% derived using data from matched subjects in the INTERMACS (Interagency Registry for Mechanically Assisted Circulatory Support) registry. Patients with ongoing HeartMate 3 support after 6-months will be followed for 24 months. Patients were adults with New York Heart Association (NYHA) class IIIB or IV, or American College of Cardiology/American Heart Association Stage D heart failure, EF < 25%, CI < 2.2 L/min/m2 without inotropes, or were inotrope-dependent on optimal medical management, or listed for heart transplant. All patients meeting the study criteria were included regardless of the indication for LVAS support: BTT and DT. Follow-up assessments include quality of life (QOL; European Quality of Life Questionnaire 5 level); functional status (six-minute walk test, NYHA functional class); adverse event rates; incidence of device malfunction, reoperation, rehospitalization, and survival free of debilitating stroke (modified Rankin Score > 3). The INTERMACS adverse event definitions were used during the trial [12].

## Results

### Patients

The 50 patients were enrolled in this clinical trial from June 2014 to November 2014, with the 6-month follow-up completed on May 26, 2015, and the 1-year follow-up completed on November 26, 2015. Details of the study design and results from the first 6 months have been previously reported (10). Table 1 lists the key demographic parameters at the time of implantation for the study group. The indication for LVAS support was nearly equally distributed between BTT (54%) and DT (46%). There were no patients classified as INTERMACS profile 1 or 7, while the majority of patients were either profile 3 (42%) or 4 (40%).

**Table 1:** Baseline demographics for the 50 trial patients

Age, years	59 ± 13
Male	90%
Indication, n (%)	
Bridge to transplantation	27 (54%)
Destination therapy	23 (46%)
INTERMACS profile	
Profile 2	5 (10%)
Profile 3	21 (42%)
Profile 4	20 (40%)
Profile 5	3 (6%)
Profile 6	1 (2%)
Inotropes	29 (58%)
Previous sternotomy	10 (20%)

### Outcomes at 1 year

**Fig. 1:**
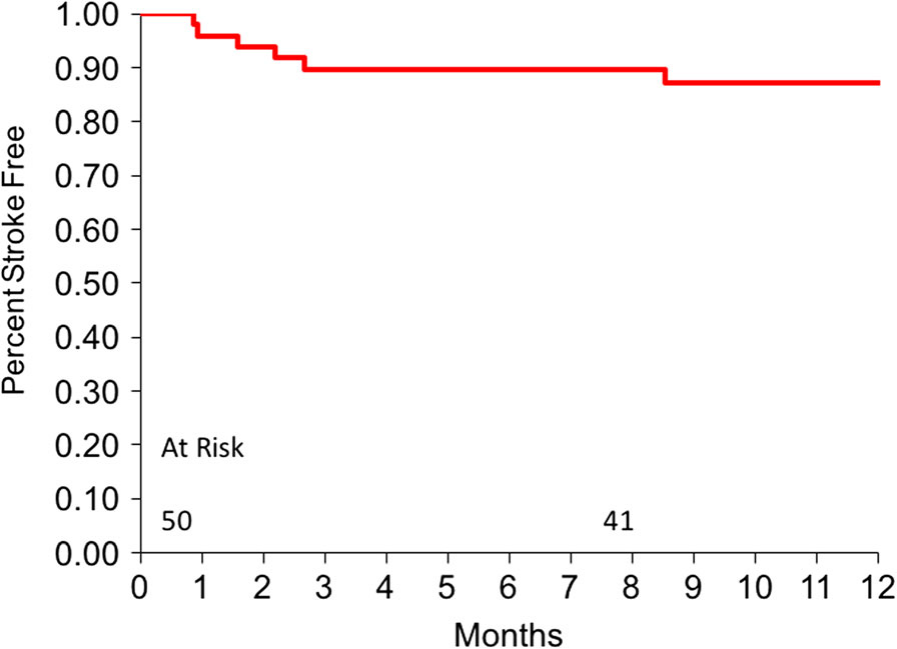
Kaplan-Meier survival to 1-year after implantation

At the 1-year follow-up, 37 (74%) patients had ongoing support. Three patients (6%) underwent transplant following 50, 101, and 231 days of support, with all alive and well at follow-up. The Kaplan-Meier survival at the 12-month time point was 81 ± 6% (Fig. 1). One patient (2%) had the device explanted on support day 185 due to sepsis and an abdominal wall abscess. There were a total of 9 deaths (18%): 4 in the first 6-months and 5 in the second 6-month follow-up time. The causes of deaths in the first 6-months were anaphylaxis, cardiac arrest, suicide and renal failure [10]. Deaths occurring during months 6 to 12 were caused by Merkel Cell carcinoma (day 203), ischemic stroke (day 212), intracerebral bleeding with international normalized ratio (INR) > 6 (day 232), and 2 cases of chronic infection with multiple organ failure (days 277 and 319).

Forty-three (86%) patients were discharged from the hospital with LVAS support. Seven patients were not discharged due to death during the index hospitalization (5) and heart transplantation (2). Twenty-eight patients (56%) had a total 66 readmissions to the hospital due to various adverse events (51; 77%), routine or scheduled testing (5), low flow alarm (2), driveline infection (1), ICD therapy (1), epistaxis (1), automobile accident (1), broken arm from fall (1), transplant evaluation (1), anticoagulation adjustment (1), and suspected device malfunction (1).

### Adverse events

**Fig. 2:**
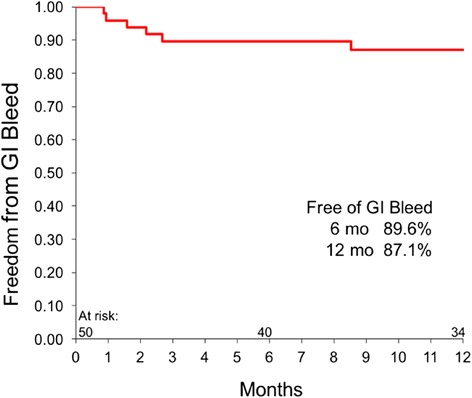
Freedom from gastrointestinal (GI) bleed

Adverse events are presented in Table 2 by the interval of occurrence (days 0 to 180 or days 181 to 365) and the total for the 1-year follow-up. Bleeding and infection were the predominate events overall, occurring in 44% and 36% of patients, respectively. Bleeding events requiring reoperation or blood transfusion were observed mostly early after implantation. The overall bleeding event rate declined from 38% in the first 6 months to 18% in the second 6-month period. The total number of patients with GI bleeding was 6 (12%), with only 2 patients (5%) experiencing this event during the second 6 months of support. The freedom from GI bleeding event was 89.6% at 6 months and 87.1% at 1 year (Fig. 2). The number of total infection events were fewer during the second 6 months (28 vs 10), with 3 (7%) having driveline infection and 2 (5%) with sepsis.

**Table 2:** Adverse events

Event	Days 0–180 (*n* = 50)			Days 181–365 (*n* = 44)			Total-1-year (*n*n = 50)		
	No. Pts	% Pts	No. Events	No. Pts	% Pts	No. Events	No. Pts	% Pts	No. Events
Bleeding	19	38	35	8	18	9	22	44	43
Requiring surgery	7	14	8	3	7	3	8	16	11
Gastrointestinal	4	8	6	2	5	3	6	12	9
Any infection	18	36	28	9	20	10	24	48	38
Sepsis	8	16	8	2	5	2	10	20	10
Driveline	5	10	5	3	7	3	8	16	8
Stroke	6	12	6	3	7	3	9	18	9
Ischemic	4	8	4	1	2	1	5	10	5
Hemorrhagic	2	4	2	2	5	2	4	8	4
Neurologic dysfunction^a^	4	8	4	0	0	0	4	8	4
Right heart failure	5	10	5	0	0	0	5	10	5
Requiring RVAD	2	4	2	0	0	0	2	4	2
Pump malfunctions	0	0	0	0	0	0	0	0	0
Pump thrombosis	0	0	0	0	0	0	0	0	0
Ouflow graft thrombosis	0	0	0	1	2	1	1	2	1
Hemolysis	0	0	0	0	0	0	0	0	0

^a^Transient ischemic attack (*n* = 2, 4%), seizure (*n* = 2, 4%)

*RVAD* right ventricular assist device

**Fig. 3:**
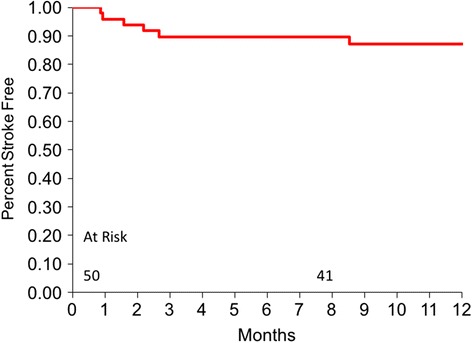
Freedom from stroke

Stroke events occurred in 6 (12%) patients in the first 6-months and in 3 (6%) patients in the second 6 months. The freedom from stroke was 87.7% at 6 months and 81.2% at 1-year (Fig. 3). Of those patients with stroke in the second 6 months, one patient experienced ischemic stroke with a modified Rankin Score (MRS) > 3 on support day 209 following rapid onset of sepsis; computed tomography scan identified outflow thrombosis, which embolized during transport to the operating room. Two patients had hemorrhagic strokes, with MRS > 3: one on support day 231 that was associated with an INR > 6, and another on day 309 that was associated with a chronic driveline infection.

During the 1-year follow-up time, there were no incidences of hemolysis, pump thrombosis or pump malfunction. Other neurologic events and right heart failure were not observed in the second 6-month follow-up.

### Functional status and quality of life

**Fig. 4:**
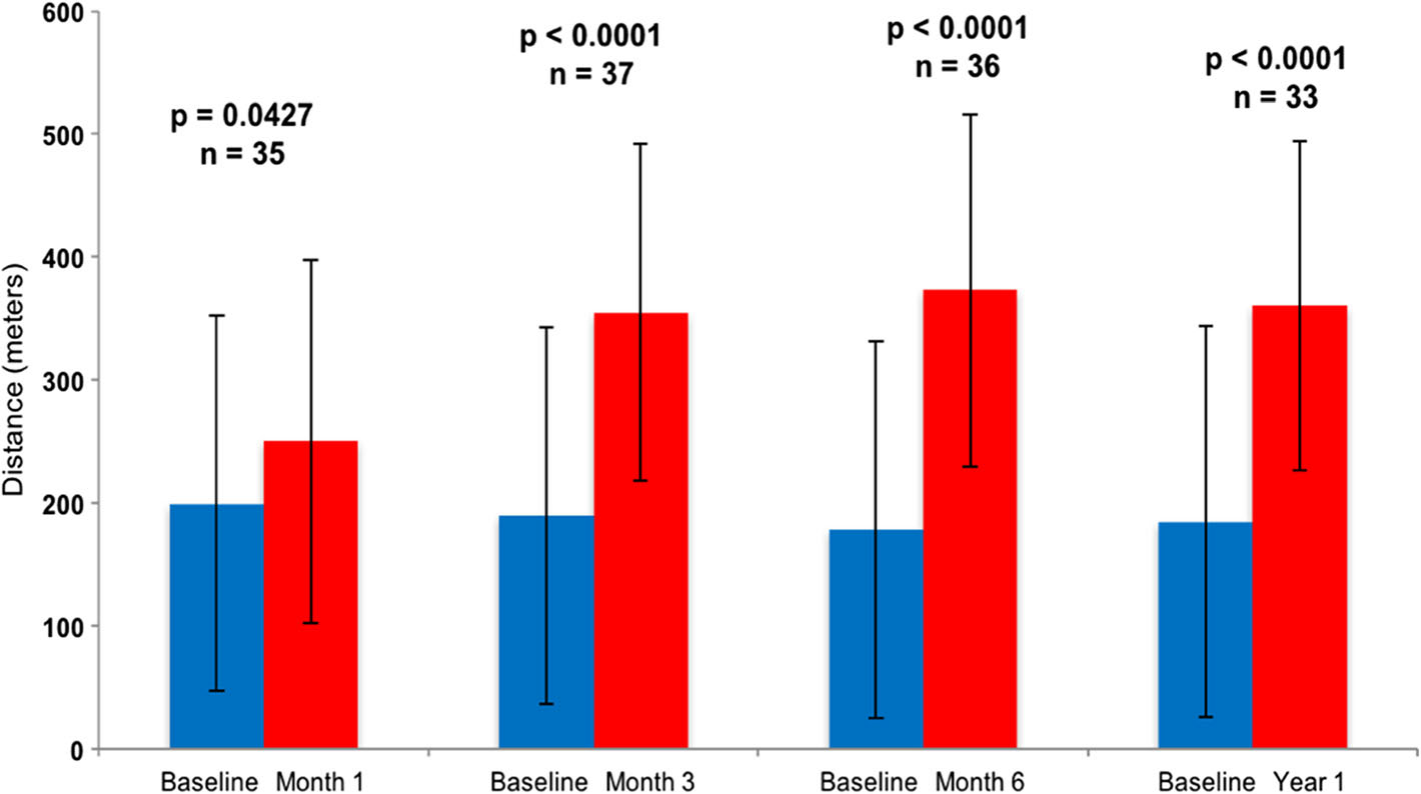
Six-minute walk test data paired for patients with results at baseline and at the specified interval

**Fig. 5:**
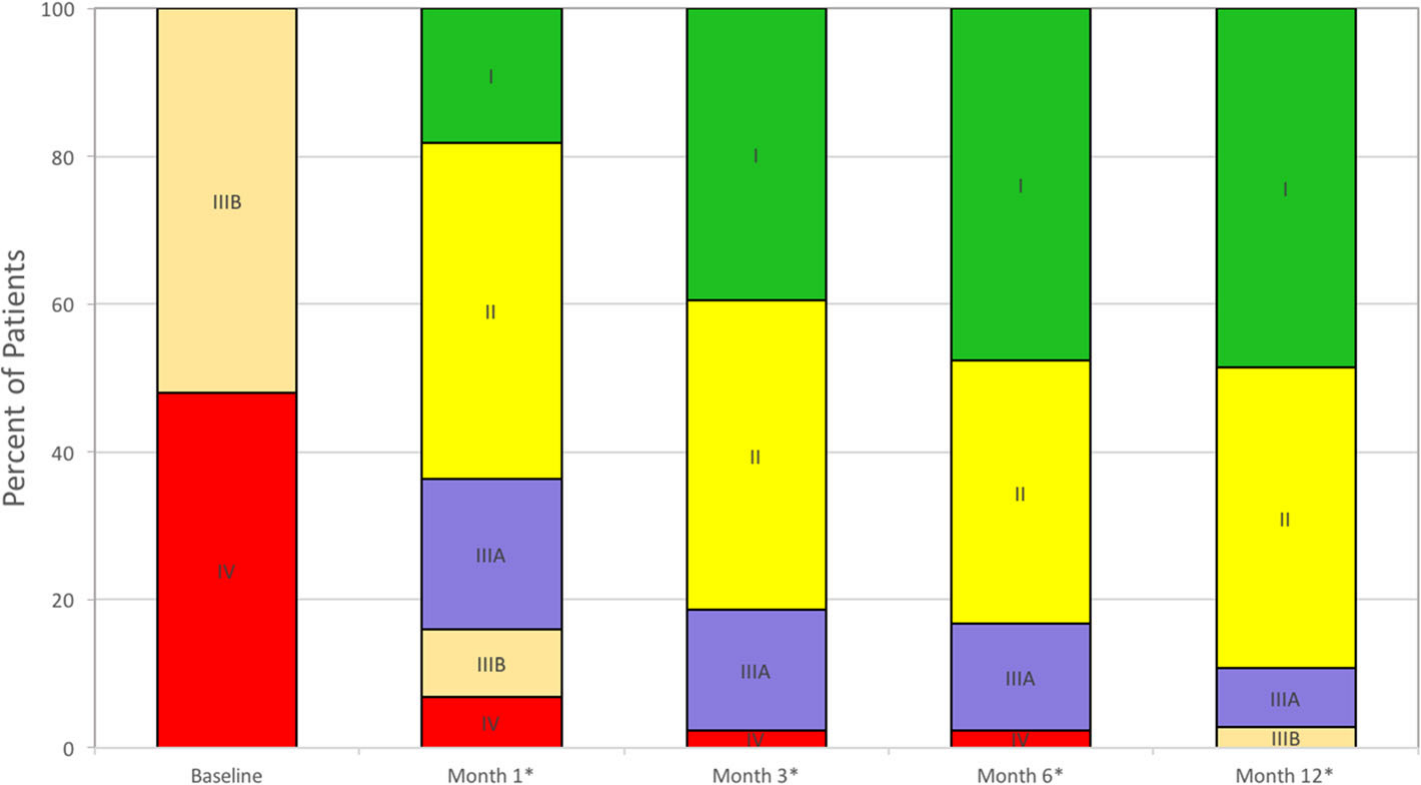
Change in the percent of patients in each NYHA classification at each interval

**Fig. 6:**
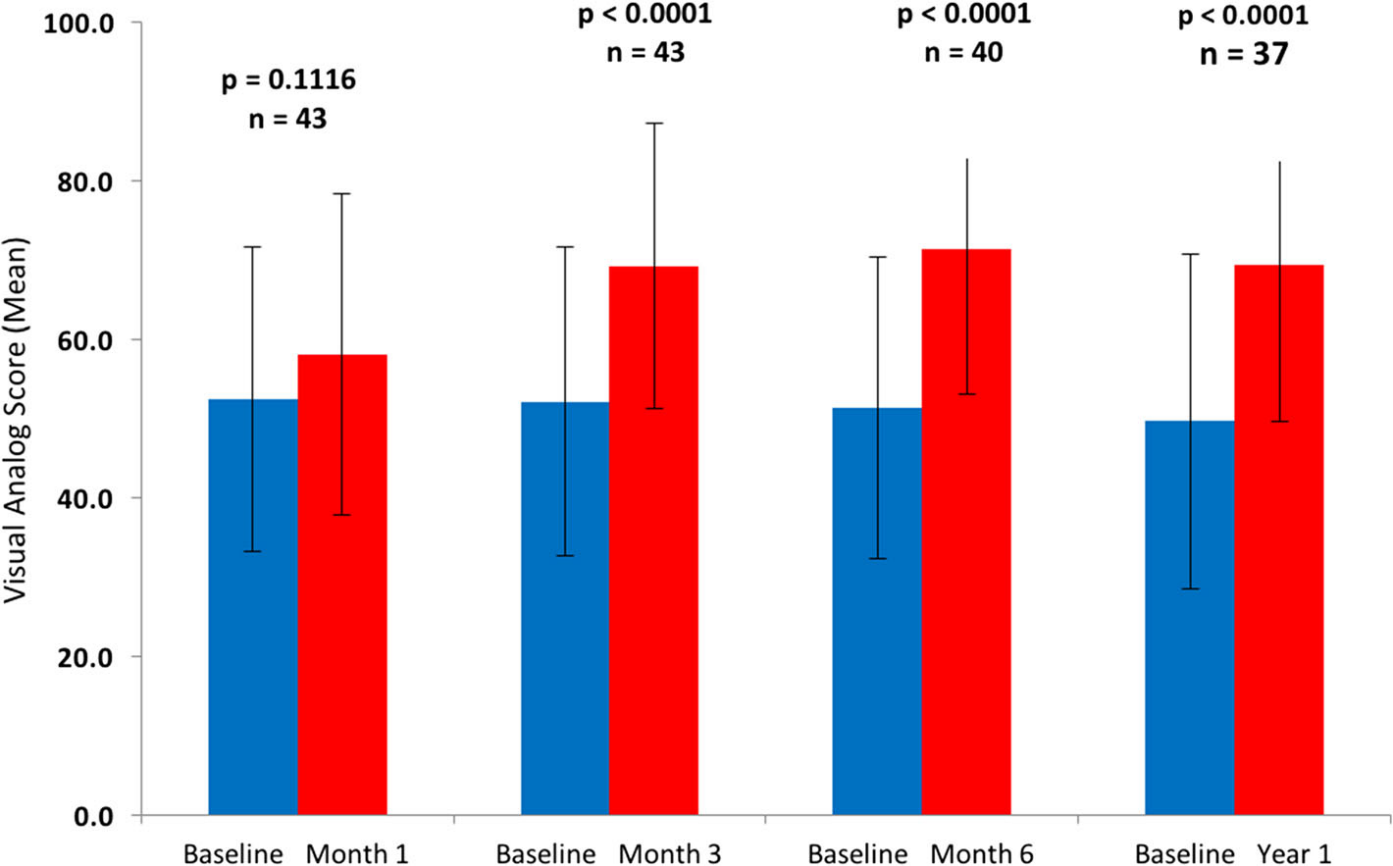
Visual analog score data paired for patients with results at baseline and at the specified interval

Patients who were able to complete the six-minute walk test before implantation and at 1, 3, 6, and 12 months showed a statistically significant improvement in walk distance at each interval (Fig. 4). At 1 year, the median distance walked was 383 m, which was an improvement of 219 m over the baseline values. The NYHA class was significantly improved (*p* <0.0001), with 89% of patients being classified as I or II at 1-year time point compared to 0% prior to implant (Fig. 5). Patient assessment of their QOL by the EuroQol visual analog score showed significant improvement at 3, 6, and 12 months (*p* <0.001) (Fig. 6).

## Discussion

This follow-up evaluation of heart failure patients supported by the HeartMate 3 for BTT and DT indicates sustained improvement in physical functioning and QOL out to 1 year. Adverse event rates tended to be lower in the second 6 months and there were no cases of hemolysis, pump thrombosis, or pump failure in the year of support. These results demonstrate comparable reliability and safety of the HeartMate 3 at 1 year of support as compared with currently available CF-LVADs.

The occurrence of GI bleeding during LVAS support is an important concern because of the need for rehospitalization and the considerable increase in the cost of care. The reported rates of GI bleeding range from 15%[13] to as high as 40%,[14] with the majority of studies reporting rates in the range of 20–30% [15–20]. Loss of von Willebrand multimers due to high shear stress and decreased arterial pulsatility are believed to be the principle characteristics of CF-LVADs that cause GI bleeding [21–24]. The 1-year rate of GI bleeding in this study was 12%, with only 5% occurring during the second half of the year. This lower rate of GI bleeding appears to be due to better preservation of von Willebrand multimeres that has been observed in patients supported by the HeartMate 3 [25].

Pump thrombosis has become a considerable concern in recent years due to an increase in incidence and the considerable morbidity associated with the event [26, 27]. Most patients survive this event by undergoing device exchange, or in a few cases thrombolytic therapy is successful. Pump exchange can be performed safely; however, there is some mortality due to major surgery and the hospital time increases cost considerably [28–31]. The rate of pump thrombosis for patients implanted with the HeartMate II LVAS (St. Jude Medical Inc., St. Paul MN) is between 6% and 12% at one year and for the HeartWare HVAD (HeartWare International Inc., Framingham, MA), the reported rate is 8% [26, 32, 33]. An important observation from this relatively small study is that there were no cases of HeartMate 3 pump thrombosis, and no pump failures, and thus no patients required pump exchange.

The overall rate of stroke at 18%, with near equal distribution between ischemic (10%) and hemorrhagic (8%) stroke, was not expected given the absence of hemolysis and pump thrombosis indicating that these events may be related other factors including blood pressure and other variables. In comparison, in recent publications the stroke rate for patients implanted with the HeartMate II LVAS (St. Jude Medical Inc., St. Paul MN) is between 10.9% and 12.1% (6 month and 2 year follow-up rate, respectively) [34, 35] and for the HeartWare HVAD (HeartWare International Inc., Framingham, MA), the 2 year follow-up reported rate is 29.7% [35]. Six (12%) of the stroke events occurred within the first 6 months and 3 (7%) occurred in the second half of the follow-up time. Factors associated with the stroke events were procedural (3), infection (3), INR > 6 (1), multiple organ failure (1), and 1 unidentified. Strokes associated with procedures were 1) difficulty placing the inflow conduit, 2) anaphylaxis from contrast media for lung CT scan, 3) following transcather aortic valve placement [10]. All patients were receiving warfarin anticoagulation, with a target INR range of 2.0–3.0, and aspirin 81–100 mg daily. Procedural-related stroke events are controllable and infection reduction efforts are continuous. Along with refined anticoagulation protocols that should be revealed in ongoing studies, there should be optimism that stroke events will decline in the future. Other neurologic events encountered were minimal, with 2 (4%) transient ischemic attacks (TIA) and 2 (4%) seizures; all which occurred within the first 6 months.

The 30-day, 6-month, and 12-month survival rates for this cohort were 98, 92, and 81%, respectively. Careful consideration must be given when comparing these results to other clinical studies with durable CF-LVADs because of the mix of BTT and DT indications. The 1-year survival rate for BTT studies is 85%[3–5]; for DT, the rate is 74% [36]. In a recent report of a study involving all patients receiving durable support (*N* = 5942) from the International Society for Heart and Lung Transplantation Mechanically Assisted Circulatory Support (IMACS) registry, the 6-month and 1-year survival rates were 86 and 80%, respectively [37]. Although any comparison to other studies is imprecise, the survival rates for this study are similar to other experiences.

### Limitations

The foremost limitations of this study was the non-randomized and non-controlled design and the relatively small number of patients in the study. Comparison to other clinical studies is not equivalent due to the mix of BTT and DT indications. With implantations at 10 different centers and in different parts of the world, patient care practices may vary by institutional preferences. All sites participating in the trial received the same training and support, and each site had similar longstanding experience with mechanical circulatory support systems.

## Conclusions

The survival rate is acceptable in this first in human series of patients with the HeartMate 3 LVAS. Major complications such as bleeding, infection, stroke, and right heart failure declined in the second 6 months of follow-up. Improvements in NYHA class, six-minute walk distance, and QOL measures demonstrate excellent circulatory support.[38] The absence of hemolysis and pump thrombosis for the 1-year follow-up is encouraging. The results observed in this study mirror that of a large randomized clinical trial just recently completed in the United States [34, 39]. In this trial that compared the HeartMate II and the HeartMate 3 in an “all-comers” design, reoperation due to pump thrombosis favored the new centrifugal flow HeartMate 3. Both HeartMate 3 studies indicate that there appears to be enhanced durability and hemocompatibility for long-term support with the HeartMate 3.

## Abbreviations

BTT: Bridge to transplant
CF-LVAD: Continuous-flow left ventricular assist device
CI: Cardiac index
DT: Destination therapy
EF: Ejection fraction
GI: Gastrointestinal
IMACS: International society for heart and lung transplantation mechanically assisted circulatory support
INR: International normalized ratio
INTERMACS: Interagency registry for mechanically assisted circulatory support
LVAS: Left ventricular assist system
NYHA: New York Heart Association
QOL: Quality of life
RVAD: right ventricular assist device
